# Separative and Comprehensive Effects of Grain Coarsening and Grain Refinement of Ni-38Cr-3.8Al Alloy during Thermal Deformation Process

**DOI:** 10.3390/ma17091965

**Published:** 2024-04-24

**Authors:** Guozheng Quan, Yifan Zhao, Qi Deng, Mingguo Quan, Yanze Yu, Daijian Wu

**Affiliations:** 1 Chongqing Key Laboratory of Advanced Mold Intelligent Manufacturing, School of Material Science and Engineering, Chongqing University, Chongqing 400044, China; z1147964348@163.com (Y.Z.); dengqi77929@sina.com (Q.D.); quanmg2258@sina.com (M.Q.); yyz6201314@sina.com (Y.Y.); wdjxhu@163.com (D.W.); 2 Jiangsu Yutaida Industrial Technology Co., Ltd., Taizhou 225300, China; 3 Huan Ding Intelligent Technology (Suzhou) Co., Ltd., Suzhou 215000, China; 4 Sichuan Laboratory of Advanced Manufacturing Technology of Press Engine, Sichuan Polytechnic University, Deyang 618000, China

**Keywords:** nickel-based alloy, grain growth, dynamic recrystallization, grain coarsening, grain refinement, kinetics model

## Abstract

During thermal deformation, grain coarsening due to grain growth and grain refinement resulting from dynamic recrystallization (DRX) collectively influence the deformed grain size. To investigate the separative and comprehensive effects of the two mechanisms in the Ni-38Cr-3.8Al alloy, grain growth experiments and isothermal compression tests were conducted. Kinetics models for grain growth and DRX behaviors were established based on the experimental data, which were integrated with finite element (FE) techniques to simulate the evolution of grain size throughout the entire thermal compression process. The effects of grain coarsening and grain refinement during this process were separated and quantified based on the simulation data. The results revealed that grain coarsening predominated during the heating and holding stages, with a longer holding time and higher holding temperatures intensifying this effect. However, during the compression stage, grain coarsening and grain refinement co-existed, and their competition was influenced by deformation parameters. Specifically, grain refinement dominated at strain rates exceeding 0.1 s^−1^, while grain coarsening dominated at lower strain rates (<0.1 s^−1^) and higher deformation temperatures (>1373 K). The simulated grain sizes closely matched the experimental observations.

## 1. Introduction

Ni-38Cr-3.8Al, a nickel-based superalloy with exceptional strength and corrosion resistance at elevated temperatures, is extensively employed for manufacturing critical components in aerospace and maritime applications [1,2]. To achieve optimum performance, the critical components made of this alloy are conventionally produced via thermal forming processes [3,4]. The grain size is a crucial indicator of microstructures which significantly impacts the material performance. Having a small grain size improves the serving performance related with grain boundary, and furtherly, it is commonly considered as the essential pursuit of a thermal forming process [5]. During such a process, the grain size of a deformed billet is dynamically determined by the comprehensive effect of two mechanisms including grain refinement induced by dynamic recrystallization (DRX) and grain coarsening induced by grain growth. However, the evolution behaviors of this comprehensive effect are highly non-linear with deformation parameters. Thus, it is meaningful to have a profound understanding of the inner mechanisms.

In a thermal deformation process, the mathematical modeling of DRX and grain growth behaviors is a necessary approach to predict and even adjust the microstructures. It is accepted that a thermal compression process comprises heating, holding, and compression stages. In the three stages, both grain growth and DRX behaviors exist, but one of them predominates at a certain time. The comprehensive effect of the two behaviors governs a complicated microstructure evolution. During the heating and holding stages, the holding temperature and time significantly impact the microstructural morphologies. Under certain holding temperatures and holding times, an uneven degree of grain growth may occur, leading to the formation of mixed grains [6]. During the compression stage, DRX is thermally activated by the accumulation of dislocation density [7]. The degree of DRX has a complex dynamic response to deformation parameters. Under conditions of relatively low deformation temperatures and moderate strain rates, the low rate of grain boundary movement and inadequate strain storage energy may result in incomplete DRX, leading to mixed grain formations [8,9]. Under conditions of relatively high deformation temperatures and low strain rates, grain growth dominates, leading to the formation of coarse grains [10]. On the other hand, at relatively low deformation temperatures and high strain rates, DRX dominates, leading to the formation of the desired fine grains [11]. In order to quantify the influence of the deformation parameters on the comprehensive effects of the two behaviors, it is essential to model the kinetics of DRX and grain growth behaviors.

Numerous studies have shown that grain growth behavior is related to holding temperature and holding time. To characterize this relationship, most studies developed grain growth kinetics models by observing the variation in grain size concerning holding temperature and holding time. Jiao et al. [12] developed a grain growth kinetic model for fine-grained extruded Mg-Nd-Zn-Zr alloys based on the Sellars equation, and their model showed that the grain growth rate is closely related to the experimental temperature. Meng et al. [13] employed the Sellars equation to model austenite grain growth in low-carbon high-strength steel. Their findings indicated that the grain growth rate of this material is slow below 1273 K, but above this temperature, the grains coarsen rapidly. Quan et al. [14] conducted a series of grain growth experiments at various holding temperatures and holding times to establish Sellars and artificial neural network models for the Ni80A superalloy, and their results showed a positive correlation between grain size and both holding time and holding temperature. Han et al. [15] utilized optical and transmission electron microscopes to investigate the grain growth behavior of manganese martensitic wear-resistant steel, analyzing the microstructural morphology and grain size across varying heating temperatures and holding times. Their subsequent development of a Sellars model, incorporating a time index, exhibited a high prediction accuracy of grain size. Guo et al. [16] examined the microstructure of Fe-15Mn-10Al-5Ni-0.8C low-density duplex steel following heat treatment, revealing δ-ferrite phases and coarse austenite dendrites as the predominant components. They subsequently developed a dynamics model to describe the growth of austenite grains and determined that the critical temperature for rapid growth is 1250 °C. Razali et al. [17] studied austenite grain growth in low-alloy steel through isothermal heat treatment. They utilized a kinetics model to predict grain size changes and optimize the model parameters with the GRG optimization method, achieving accurate predictions. In summary, the grain growth kinetics models established in the above studies accurately described the grain coarsening effect at elevated temperatures.

The DRX behavior is notably influenced by deformation parameters, including temperature, strain rate, and strain [18]. Many researchers established DRX dynamics models through the results of thermal compression tests to describe the dynamic response of DRX to deformation parameters [19,20,21]. The established models are often integrated with finite element (FE) methods to present the microstructure evolution more intuitively. Such studies have been carried out on nickel-based alloys. Li et al. [22] investigated the DRX behavior of a nickel-based superalloy under thermal deformation conditions, finding that elevated deformation temperatures lead to increased DRX grain size and DRX volume fraction. Quan et al. [23] integrated the established DRX kinetics models for the Ni80A superalloy into an FE platform for predicting grain size evolution, and their predicted grain sizes closely matched the experimental values. Chen et al. [24] accurately forecasted the deformed grain size of a nickel-based superalloy during thermal compression by combining DEFORM-3D V10.2 software with DRX dynamics models, and they found significant grain refinement at relatively high strain rates and low temperatures. Hara et al. [25] studied the microstructure evolution of a nickel-cobalt based superalloy during hot forging and constructed a DRX grain size prediction model, which had a prediction error within 12%. Geng et al. [11] conducted an analysis and modeling of DRX kinetics to study the changes in microstructure of the GH4169 superalloy. They found that at higher temperatures and lower strain rates, there was a significant rise in the degree of DRX. The above studies show that the combination of FE techniques with the DRX kinetics model can accurately predict the microstructural evolution during deformation. 

The grain size evolution during thermal deformation is determined by both grain growth and DRX behaviors. However, most studies have only focused on one of these behaviors, which cannot accurately describe the evolution of grain size throughout a complete thermal deformation process that includes heating, holding, and deformation stages. Thus, there is a need for quantitative analysis that comprehensively considers both the grain coarsening and grain refinement effects. In the present study, grain growth experiments and isothermal compression tests were conducted on the Ni-38Cr-3.8Al alloy. The grain growth and DRX behaviors were investigated through kinetics modeling based on the experimental data. Subsequently, an FE model was developed to predict the evolution of the comprehensive effects of grain coarsening and grain refinement. The respective effects of grain growth and DRX on grain size were separated and quantified using the simulation data. The accuracy of the model was verified by comparing its prediction values with experimental ones. This research provides insights into the comprehensive effects of grain coarsening and grain refinement in the Ni-38Cr-3.8Al alloy, aiding in predicting and adjusting microstructures during the thermal deformation process.

## 2. Experiments and Model Equations

### 2.1. Experiments

**Figure 1:**
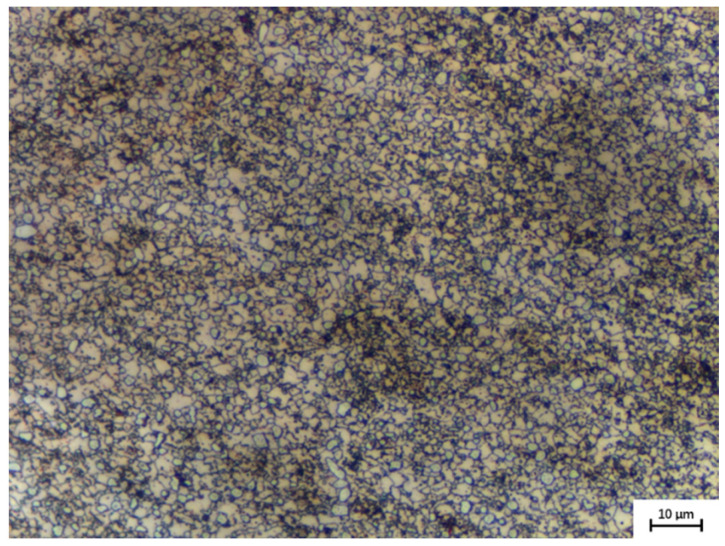
The initial microstructure of Ni-38Cr-3.8Al alloy.

**Figure 2:**
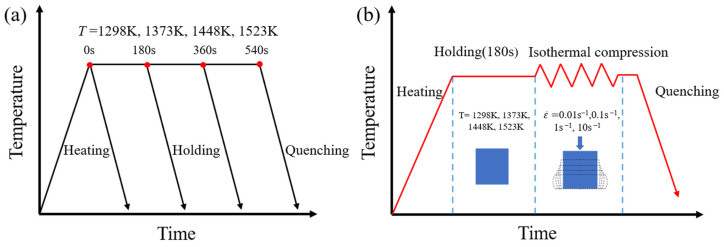
The procedures of (**a**) the grain growth experiment and (**b**) the isothermal compression test.

The experimental material is the Ni-38Cr-3.8Al alloy with a chemical composition (wt.%) of Cr-39.1, Al-3.8, C-0.02, B-0.003, and Ni-balance. The initial microstructure of this alloy is shown in Figure 1, with an average grain size of 3.48 µm. To explore grain growth behavior, sixteen cylindrical specimens, each measuring 12 mm in height and 10 mm in diameter, were prepared from a single forged billet. The grain growth experiment followed the sequence illustrated in Figure 2a. These specimens were divided into four groups, with each group consisting of four specimens. The four groups correspond to holding temperatures of 1298 K, 1373 K, 1448 K, and 1523 K, respectively. Each group was heated in a numerically controlled furnace at a rate of 5 K/s until reaching the designated holding temperature. At 0 s, 180 s, 360 s, and 540 s into the holding stage, one specimen was taken out from each group and immediately quenched in water. Concurrently, sixteen cylindrical specimens of the same size as before were prepared from the same billet for isothermal compression tests. The tests were performed utilizing a Gleeble-3500 thermal simulator and included four distinct strain rates (0.01 s^−1^, 0.1 s^−1^, 1 s^−1^, and 10 s^−1^) and four different temperatures (1298 K, 1373 K, 1448 K, and 1523 K). The procedure, outlined in Figure 2b, included heating the specimens to the designated deformation temperature at a rate of 5 K/s, followed by a 180 s hold. Compression was applied until a true strain of 0.916 (60% reduction in height) was attained, using a specified constant strain rate. Water quenching immediately after compression was carried out to preserve high-temperature microstructures. It is worth noting that the temperature control deviation of both the numerically controlled furnace and the Gleeble-3500 thermal simulator (DSI, Nashville, TN, USA) does not exceed 5 K.

For microstructure characterization, the heated specimens were cut in half horizontally, while the compressed ones were cut along the compression axis. Afterward, the section surfaces were ground, polished, and corroded by an etching solution prepared from 1 g KMnO_4_, 10 mL H_2_SO_4_, and 90 mL H_2_O. Microstructural observations were then carried out at the center of each section surface using an optical microscope. 

Notably, the four temperatures used in the grain growth experiment and the isothermal compression test were identical, and the heating rates during the heating stage were consistent. Additionally, the holding time in the grain growth experiment was sufficient to cover the entire isothermal compression process. This ensured an accurate kinetics description of both grain growth and DRX behavior within the same thermal compression process.

### 2.2. Model Equations

The Sellars model was employed in this study to characterize the grain growth behavior of the Ni-38Cr-3.8Al alloy [26]. The model is described as follows.

(1) $$d^{m}=d_{0}^{m}+At\exp[-Q_{\mathrm{gg}}/(RT)]$$

where *d* represents the average grain size under different holding temperatures and holding times, μm, *d*_0_ represents the initial grain size, μm, *Q*_gg_ is the grain growth activation energy, J mol^−1^, *A* and *m* are material-related constants, *R* is the universal gas constant taken as 8.31 J mol^−1^ K^−1^, *T* is the holding temperature, K, and *t* is the holding time, s.

The JMAK equation and Sellars model are commonly used to describe the DRX volume fraction and DRX grain size, respectively. The JMAK equation and the Sellars model, along with their associated terms $\varepsilon_{c}$ and $\varepsilon_{0.5}$, are represented by Equation (2) [27,28].

(2) $$\left\{\begin{array}{l} X_{\mathrm{drx}}=1-\exp\left[-\beta_{d}{\left(\frac{\varepsilon -\varepsilon_{c}}{\varepsilon_{0.5}}\right)}^{k_{d}}\right]\\ \varepsilon_{c}=a\varepsilon_{p}\\ \varepsilon_{p}=a_{1}d_{0}^{n_{1}}{\dot{\varepsilon }}^{m_{1}}\exp(Q_{1}/RT)\\ \varepsilon_{0.5}=a_{2}d_{0}^{n_{2}}{\dot{\varepsilon }}^{m_{2}}\exp(Q_{2}/RT)\\ d_{\mathrm{drx}}=a_{3}d_{0}^{n_{3}}{\dot{\varepsilon }}^{m_{3}}\exp(Q_{3}/RT) \end{array}\right.$$

where *X*_drx_ is the DRX volume fraction, ε is the true strain, $\dot{\varepsilon }$ is the strain rate, $\varepsilon_{c}$ is the critical strain, $\varepsilon_{p}$ is the peak strain, $\varepsilon_{0.5}$ is the strain corresponding to a DRX volume fraction of 50%, *T* is the deformation temperature, K, *d*_0_ is the grain size, μm, when undeformed at different deformation temperatures, *d*_drx_ is the DRX grain size, μm, *Q*_1_, *Q*_2_, and *Q*_3_ are the deformation activation energy values, J mol^−1^, and *β*_d_, *k*_d_, *a*, *a*_1_, *a*_2_, *a*_3_, *n*_1_, *n*_2_, *n*_3_, *m*_1_, *m*_2_, and *m*_3_ are material-related constants. 

## 3. Results and Discussions

### 3.1. Grain Growth Behavior during Heating and Holding Processes

#### 3.1.1. Influence of Holding Temperature

**Figure 3:**
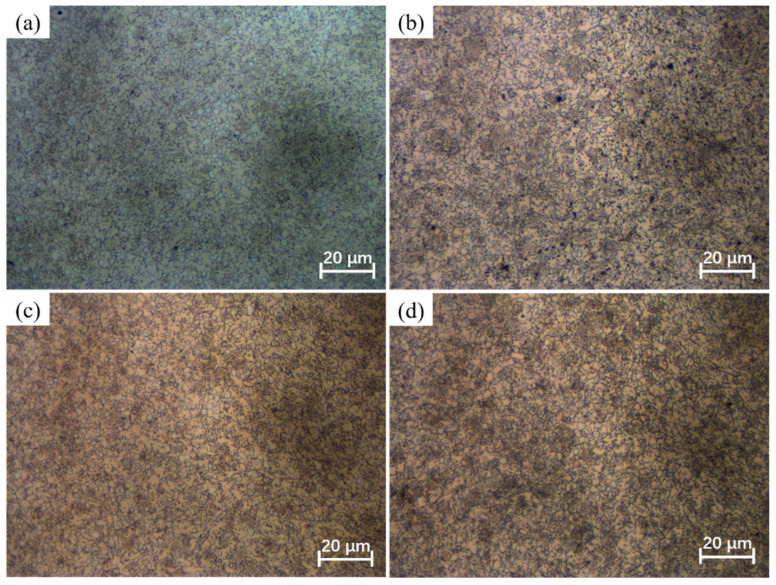
The microstructures of Ni-38Cr-3.8Al alloy heated at 1298 K for different holding times: (**a**) 0 s, (**b**) 180 s, (**c**) 360 s, (**d**) 540 s.

**Figure 4:**
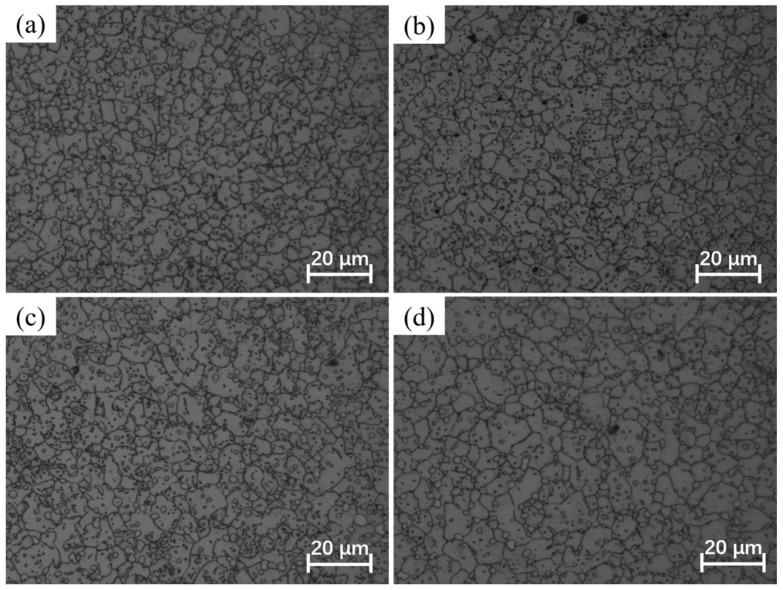
The microstructures of Ni-38Cr-3.8Al alloy heated at 1373 K for different holding times: (**a**) 0 s, (**b**) 180 s, (**c**) 360 s, (**d**) 540 s.

**Figure 5:**
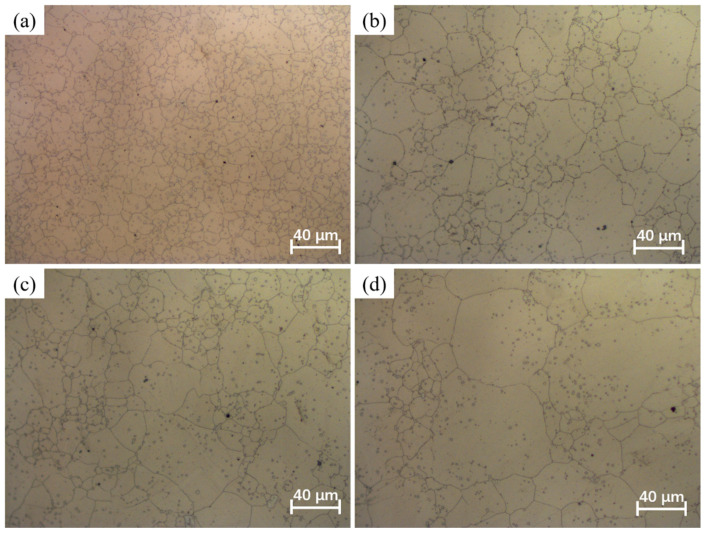
The microstructures of Ni-38Cr-3.8Al alloy heated at 1448 K for different holding times: (**a**) 0 s, (**b**) 180 s, (**c**) 360 s, (**d**) 540 s.

**Figure 6:**
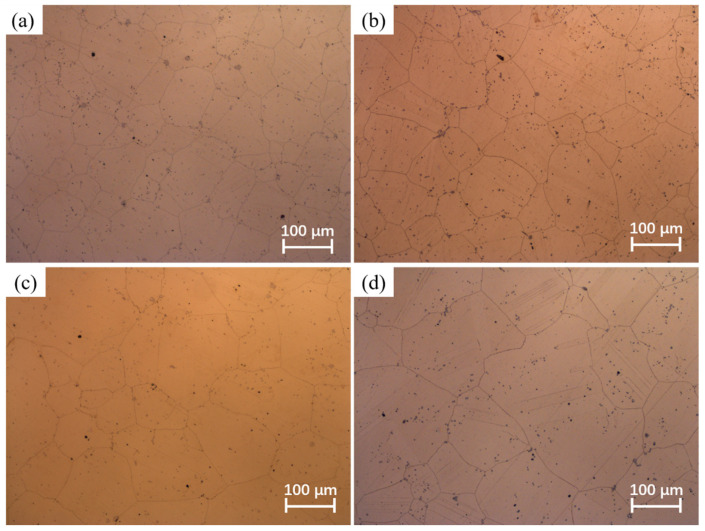
The microstructures of Ni-38Cr-3.8Al alloy heated at 1523 K for different holding times: (**a**) 0 s, (**b**) 180 s, (**c**) 360 s, (**d**) 540 s.

The phenomenon of grain coarsening is intricately linked to grain growth behavior. At elevated temperatures, the acceleration of atomic diffusion promotes grain boundary migration, leading to pronounced grain growth [29]. The holding temperature and holding time play a crucial role in influencing grain growth behavior. Figure 3, Figure 4, Figure 5 and Figure 6 shows the microstructures of the Ni-38Cr-3.8Al alloy that have been heated to temperatures ranging from 1298 K to 1523 K and held for 0 s to 540 s. At 1298 K and 1373 K, there is minimal growth in grain size, and the shape of the grains is mainly polygonal. There are also many precipitates distributed along the grain boundaries. As the heating temperature rises to 1448 K and 1523 K, the grains begin to coarsen significantly. Mixed-grain microstructures are observed at 1448 K. In addition, there is a significant decrease in the quantity of precipitates at both 1448 K and 1523 K. The average grain sizes at various holding temperatures and holding times have been statistically calculated and are presented in Table 1. 

**Figure 7:**
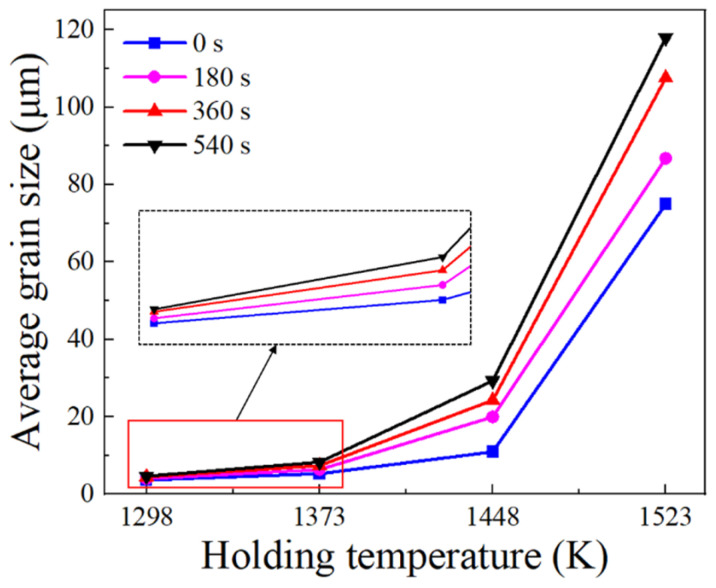
Changes in average grain size with holding temperature at different holding times.

The initial grain sizes at various temperatures ranged from 3.66 μm to 74.98 μm, with large differences. This indicates that the temperature has a significant impact on grain growth behavior. Figure 7 depicts the variations in the average grain size with holding temperatures at different holding times. Apparently, the average grain size increases with rising temperatures for each holding time, indicating a higher grain growth rate at elevated temperatures. This augmented grain growth rate is associated with the speed of grain boundary migration [30]. With higher temperatures, the atomic diffusion rate accelerates, consequently amplifying the speed of grain boundary migration. 

Additionally, the pinning pressure exerted by the precipitates on the grain boundaries can impede grain boundary migration, thereby hindering grain growth [27,31]. The holding temperature significantly affects the quantity of precipitates. From the microstructures at different holding temperatures in Figure 3, Figure 4, Figure 5 and Figure 6, it is evident that numerous precipitates exist on grain boundaries at temperatures of 1298 K and 1373 K, accompanied by smaller grain sizes. However, at 1448 K and 1523 K, there is a notable decrease in the quantity of precipitates, accompanied by a remarkable increase in grain size. It is worth noting that the microstructure exhibits a mixed-grain morphology at the holding temperature of 1448 K. This phenomenon occurs because the precipitates start to dissolve, allowing local grain boundaries to overcome the pinning effect, thereby causing specific grains to grow slightly and acquire more neighbors. This enhances the mobility of local grain boundaries, result in a self-acceleration of the single-grain growth effect. Eventually, this leads to the formation of a mixed-grain morphology [32]. At the holding temperature of 1523 K, the near-complete dissolution of precipitates enables the active growth of fine grains, ultimately resulting in a microstructural morphology with a uniform grain size [33].

**Figure 8:**
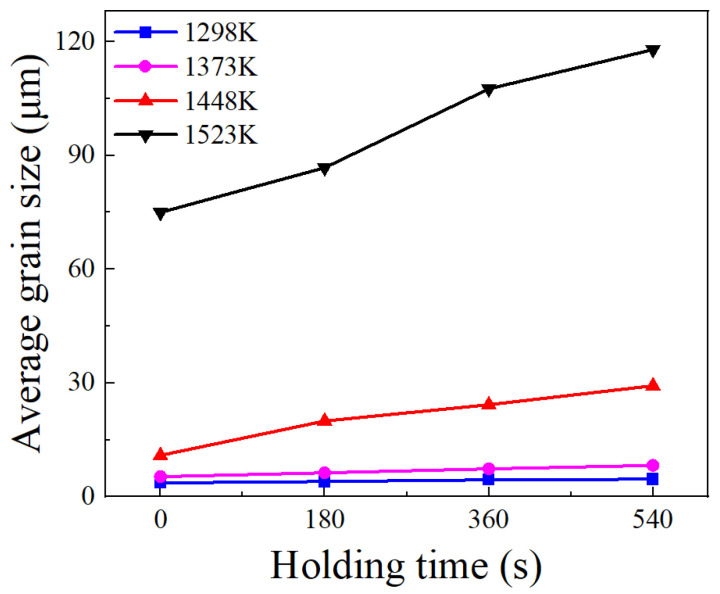
Changes in average grain size with holding time at different holding temperatures.

Holding time is another important factor influencing grain growth behavior. Figure 8 illustrates the changes in the average grain size with holding times at distinct holding temperatures. As the holding time prolongs, there is a gradual increase in the average grain size, while the grain growth rate concurrently slows down. This phenomenon is attributed to the time-consuming nature of grain boundary migration. A prolonged holding time allows enough time for grain boundary migration. However, as the holding time increases, the driving force is limited, and further migration of grain boundaries becomes difficult. Consequently, the grain growth rate slows down as the holding time increases. Moreover, the microstructures observed at various holding times in Figure 3, Figure 4, Figure 5 and Figure 6 indicate that as the holding time extends, the precipitates located at both grain boundaries and within the grains gradually dissolve. This dissolution process weakens the inhibitory effect on grain boundary migration, consequently leading to a gradual increase in grain size as the holding time increases.

#### 3.1.2. Modeling of Grain Growth Kinetics

The experimental findings suggest that the grain size is highly responsive to both the holding temperature and holding time. To further the correlation between grain size, holding time, and holding temperature, it is imperative to develop a Sellars model that accurately describes the grain growth behavior of the Ni-38Cr-3.8Al alloy. 

Taking logarithms of both sides of Equation (1) yields Equation (3).

(3) $$\ln(d^{m}-d_{0}^{m})=\ln A+\ln t-Q_{\mathrm{gg}}/(RT)$$

When the value of *m* is known and holding time *t* is fixed, grain size *d* is only determined by holding temperature *T*. From Equation (3), *Q*_gg_ can be calculated using the following equation.

(4) $$Q_{\mathrm{gg}}=-R\cdot \frac{𝜕\ln(d^{m}-d_{0}^{m})}{𝜕(1/T)}$$

This study assumed that *m* is a constant value, specifically 1, 2, 3, 4, 5, 6, 7, or 8. Subsequently, the corresponding values of *A* and *Q*_gg_ were calculated for each assumption value of *m*. Furthermore, the mean squared error (MSE) was utilized to assess the precision of Equation (1) for various *m* values. The formula to calculate the MSE is given in Equation (5).

(5) $$\mathrm{MSE}=\frac{1}{N}\sum_{i=1}^{N}{\left(E_{i}-P_{i}\right)}^{2}$$

where *E_i_* is the experimental value, *P_i_* is the predicted value, and *N* is the number of samples.

**Figure 9:**
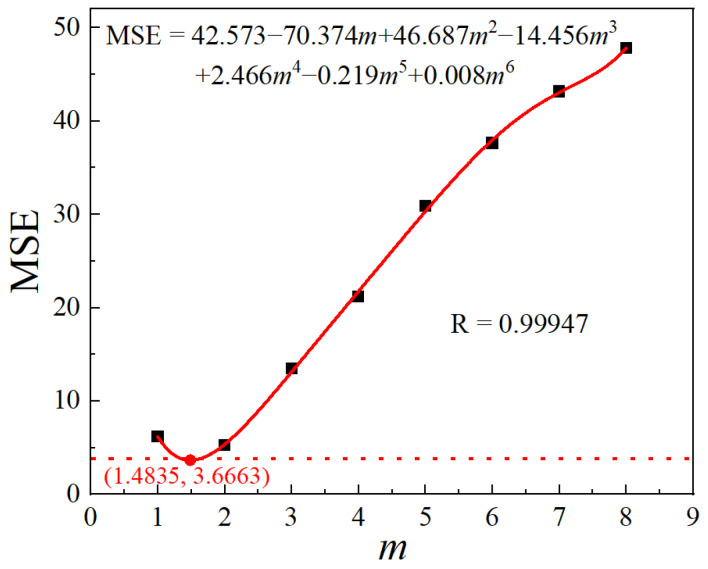
Relationship between MSE and *m*.

The values of *A*, *Q*_gg_, and MSE under different *m* values are listed in Table 2. Further, a polynomial was fitted to the relationship between MSE and *m*, as shown in Figure 9. From the fitting curve, the optimal solution for *m* in the model is *m* = 1.4835, corresponding to the minimum MSE value of 3.6663. 

**Figure 10:**
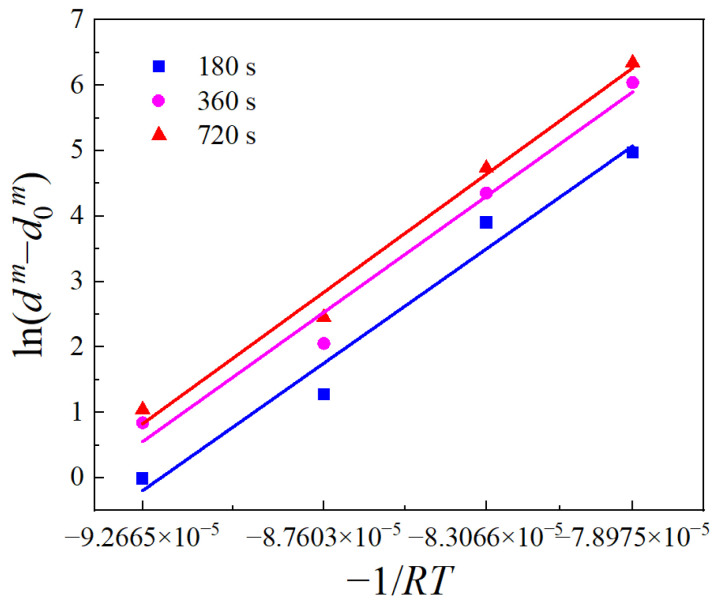
Relationships between ln(*d^m^* − *d*_0_*^m^*) and −1/(*RT*).

The relationships between ln(*d^m^* − *d*_0_*^m^*) and −1/(*RT*) are derived from Equation (3) by substituting *m =* 1.4835, as shown in Figure 10. The average slope of these fitting lines is the *Q*_gg_ value, which is calculated as 390,813.2583 J/mol. Substituting *Q*_gg_ into Equation (3) yields the *A* value at different holding temperatures and times. The average of the obtained values is taken as the final *A* value, which is 2.5252 × 10^13^.

In summary, the grain growth model of the Ni-38Cr-3.8Al alloy is obtained as follows:

(6) $$d^{1.4835}=d_{0}^{1.4835}+2.5252\times 10^{13}t\exp[-390813.2583/(RT)]$$

**Figure 11:**
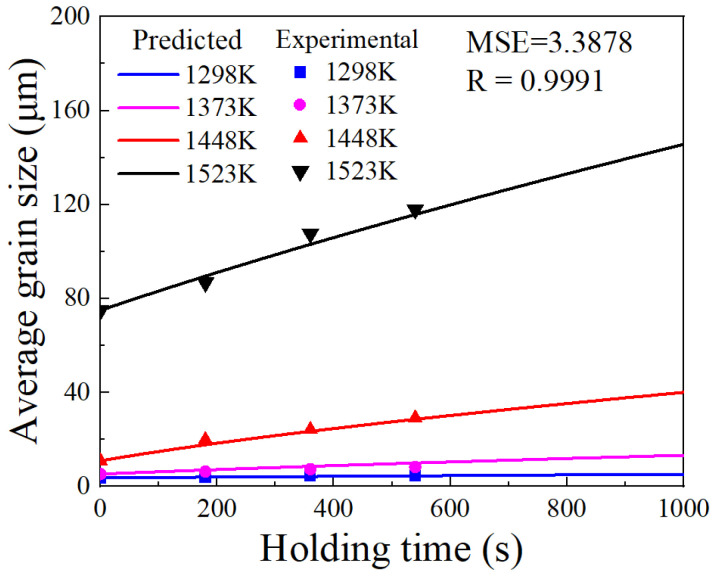
Comparison of experimental and predicted grain sizes.

To validate the established grain growth model, a comparison is made between the predicted and experimental grain sizes, as illustrated in Figure 11. The MSE for the predicted grain size is 3.3878, which closely matches the previously forecasted MSE value. The correlation coefficient (R) value is 0.9991, nearing 1. These comparative results affirm the excellent predictive capability of the established grain growth model.

### 3.2. DRX Behavior during Thermal Compression Process

#### 3.2.1. DRX Characteristics in Stress–Strain Curves

**Figure 12:**
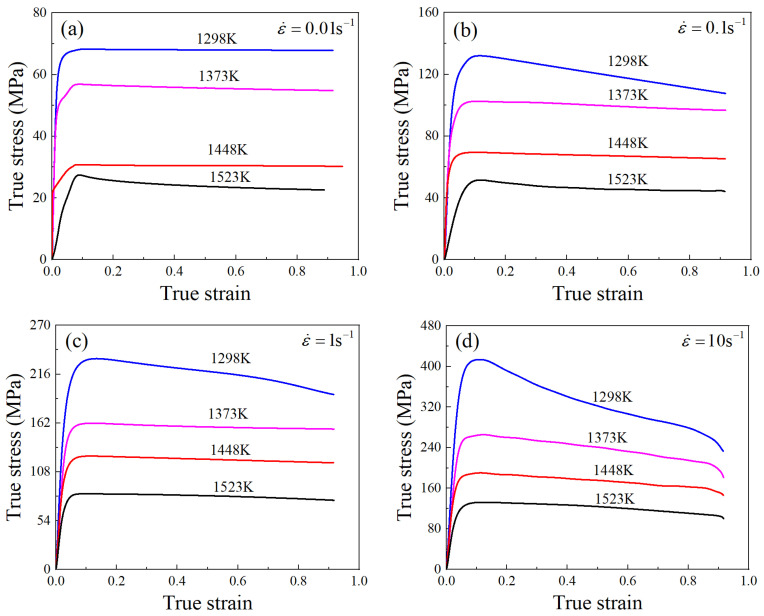
True stress–strain curves of Ni-38Cr-3.8Al alloy under temperatures of 1298–1523 K and strain rates of 0.01–10 s^−1^ [34]: (**a**) 0.01 s^−1^, (**b**) 0.1 s^−1^, (**c**) 1 s^−1^, (**d**) 10 s^−1^.

In contrast to the role of grain growth, DRX exhibits a significant grain refinement effect and serves as the main softening mechanism during thermal deformation. The true stress–strain curves of the Ni-38Cr-3.8Al alloy derived through isothermal compression tests are shown in Figure 12. The feature of DRX softening is prominently manifested in the stress–strain curves. The variation in flow stress can be divided into three stages. Initially, there is a rapid increase in flow stress with the true strain, indicating the characteristics of work hardening. During the second stage, the dynamic softening effect of DRX and dynamic recovery (DRV) gradually increases. Consequently, the flow stress exhibits a slower increase, reaching its peak when work hardening and dynamic softening achieve equilibrium. In the third stage, the DRX softening takes over, resulting in a gradual reduction in flow stress. Eventually, the flow stress stabilizes due to a dynamic equilibrium between DRX softening and work hardening. 

#### 3.2.2. DRX Characteristics in Stress–Strain Curves

**Figure 13:**
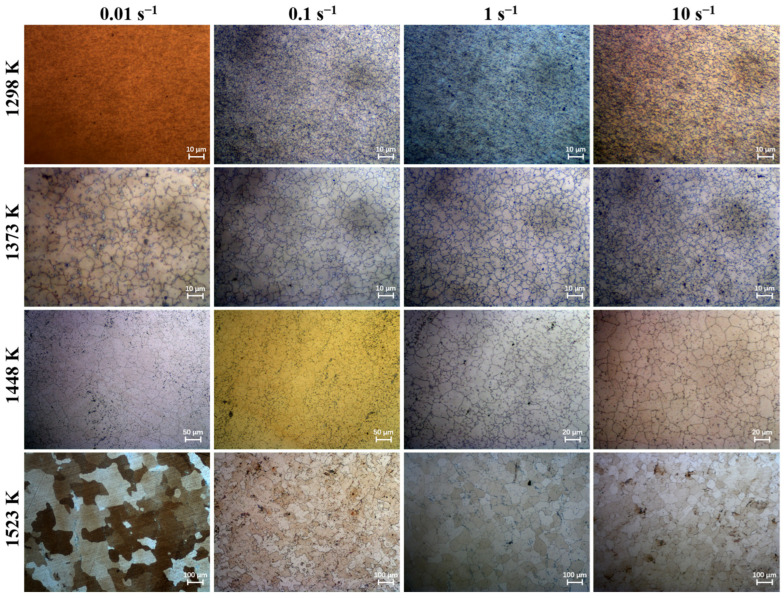
The microstructures of Ni-38Cr-3.8Al alloy under various deformation temperatures and strain rates.

The softening effect of DRX is directly associated with its grain refinement mechanism. When the alloy undergoes a large deformation, the dislocation density increases substantially. These dislocations slip, climb, and undergo polygonization, producing subgrains that gradually grow into recrystallized grains. These fine strain-free recrystallized grains entirely replace the original grains, thereby reducing the flow stress during deformation. The DRX behavior is greatly influenced by the deformation temperature and strain rate. Figure 13 displays the microstructures of deformed specimens at different deformation temperatures and strain rates. It is evident that the microstructures are predominantly comprised of equiaxed grains under all isothermal compression conditions. This observation indicates a high degree of DRX across all deformation conditions. 

Table 3 presents the average deformed grain size at various deformation temperatures and strain rates. The influence of deformation temperature and strain rate on grain size is evident. With a constant deformation temperature, there is a decreasing trend in grain size as the strain rate increases. This is because during thermal deformation, recrystallization nuclei are generated at grain boundaries and gradually develop into equiaxed grains, replacing the original grains with refined equiaxed grains. The growth of these equiaxed grains is influenced by the strain rate, where higher rates lead to smaller grains due to insufficient time for growth. Conversely, at a constant strain rate, the grain size shows an increasing trend with rising deformation temperatures. This occurrence is attributed to the elevated deformation temperature promoting the rapid migration of grain boundaries, facilitating the rapid growth of equiaxed grains generated by DRX. Ultimately, this results in larger grain sizes.

#### 3.2.3. Modeling of DRX Kinetic

1. Initiation of DRX

The DRX behavior is greatly influenced by deformation temperature, strain rate, and strain. Therefore, DRX kinetics modeling is essential for accurately describing the grain refinement effect during thermal deformation. The onset of DRX corresponds to the attainment of the critical strain *ε*_c_. The relationship between the critical strain *ε*_c_ and the peak strain *ε*_p_ can be expressed as follows:

(7) $$\varepsilon_{c}=a\varepsilon_{p}$$

(8) $$\varepsilon_{p}=a_{1}d_{0}^{\;n_{1}}{\dot{\varepsilon }}^{m_{1}}\exp(Q_{1}/RT)$$

According to Poliak et al. [35,36], the critical strain *ε*_c_ of DRX was identified at the inflection point on the fitted curve of the work hardening rate *θ* versus the flow stress *σ*. The value of *θ* was calculated from Equation (9).

(9) θ=𝜕σ/𝜕ε

where *θ* represents the work hardening rate, *σ* is the flow stress, and *ε* is the true strain. 

**Figure 14:**
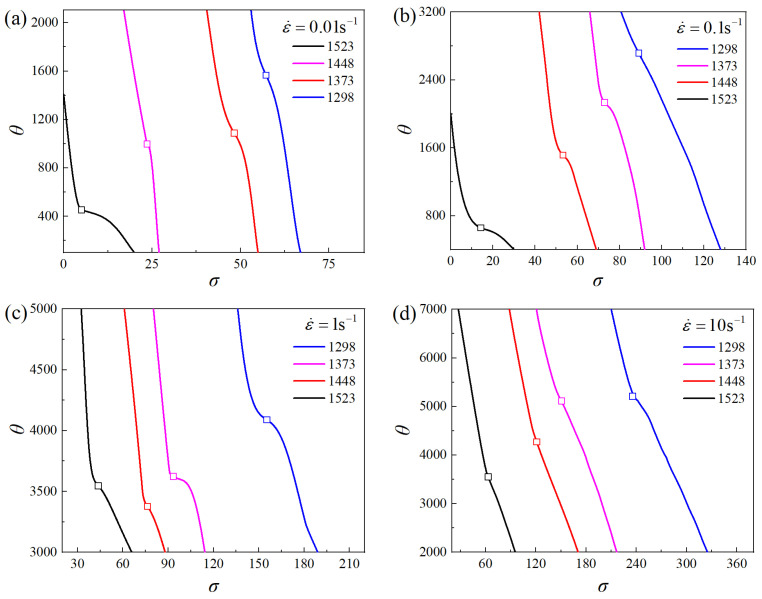
*θ*-*σ* curves under temperatures of 1298–1523 K and strain rates of 0.01–10 s^−1^: (**a**) 0.01 s^−1^, (**b**) 0.1 s^−1^, (**c**) 1 s^−1^, (**d**) 10 s^−1^.

From the true stress–strain curves, the *θ*-*σ* plots for all deformation conditions were derived, as illustrated in Figure 14. The inflection points marked on these *θ*-*σ* curves mark the start of DRX, from which the critical stress *σ*_c_ can be identified. Subsequently, the critical strain *ε*_c_ and the peak strain *ε*_p_ were determined from the true stress–strain curves and are listed in Table 4. A linear fit was applied to establish the relationship between *ε*_c_ and *ε*_p_ according to Equation (7), and the material constant *a* was obtained as 0.2831. 

Applying logarithms to both sides of Equation (8) yields Equation (10).

(10) $$\ln\varepsilon_{p}=\ln a_{1}+n_{1}\ln d_{0}+m_{1}\ln\dot{\varepsilon }+\frac{Q_{1}}{RT}\;$$

Due to the influence of the holding temperature, the specimens had different pre-deformation grain sizes at different deformation temperatures, as shown in Table 5. By the nonlinear fitting of *ε*_p_, *d*_0_, $\dot{\varepsilon },$ and *T* in Equation (10), *a*_1_ = 0.0329, *n*_1_ = −0.0023, *m*_1_ = 0.0347, and *Q*_1_ = 14,134.3081 J/mol were calculated.

In summary, the models of critical strain for the Ni-38Cr-3.8Al alloy can be expressed by Equations (11) and (12).

(11) $$\varepsilon_{c}=0.2831\varepsilon_{p}$$

(12) $$\varepsilon_{p}=0.0329{d_{0}}^{-0.0023}{\dot{\varepsilon }}^{0.0347}\exp(14,134.3081/RT)$$

2. The volume fraction model of DRX

The DRX degree during thermal deformation can be quantified using the DRX volume fraction, which is defined by Equations (13) and (14).

(13) $$X_{\mathrm{drx}}=1-\exp\left[-\beta_{d}{\left(\frac{\varepsilon -\varepsilon_{c}}{\varepsilon_{0.5}}\right)}^{k_{d}}\right]$$

(14) $$\varepsilon_{0.5}=a_{2}d_{0}^{n_{2}}{\dot{\varepsilon }}^{m_{2}}\exp(Q_{2}/RT)$$

**Figure 15:**
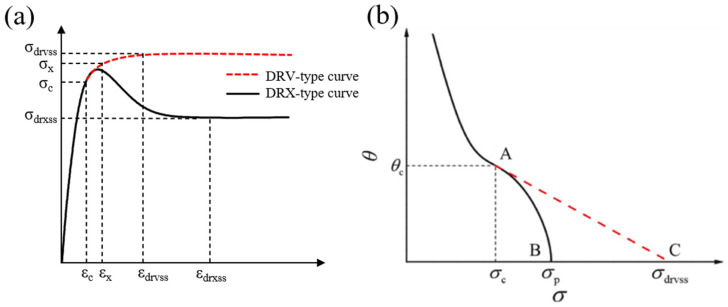
(**a**) Typical DRX and DRV curves. (**b**) Determination of *σ*_drvss_ and *σ*_drvx_ by *θ*-*σ* curve.

To compute the material constants in Equations (13) and (14), the DRX volume fractions *X*_drx_ under various deformation conditions were necessary. According to Zahiri et al. [37], *X*_drx_ can be determined from the true stress–strain curves using Equation (15).

(15) $$X_{\mathrm{drx}}=\frac{{\left(\sigma_{\mathrm{drvx}}\right)}^{2}-{\left(\sigma_{\mathrm{drxx}}\right)}^{2}}{{\left(\sigma_{\mathrm{drvss}}\right)}^{2}-{\left(\sigma_{\mathrm{drxss}}\right)}^{2}}$$

where *σ*_drvss_ and *σ*_drvx_ represent the steady-state stress and the transient-state stress on the stress–strain curves with the ideal DRV type (shown in Figure 15a), and *σ*_drxss_ and *σ*_drxx_ represent the steady-state stress and the transient-state stress on the stress–strain curves with the ideal DRX type (shown in Figure 15a). 

Two typical flow curves were classified according to the predominant softening mechanism during hot deformation, as shown in Figure 15b. The solid curve is the ideal stress–strain curve of the DRX type, from which the values of *σ*_drxss_ and *σ*_drxx_ can be obtained directly. The dashed one is the stress–strain curve with the ideal DRV type, considering only dynamic recovery. In order to ascertain the values of *σ*_drvss_ and *σ*_drvx_, a corresponding ideal stress–strain curve of the DRV type was developed, as depicted by the dashed lines in Figure 15b. In Figure 15b, point A denotes the critical point for the onset of DRX; point B denotes the point of peak stress; and point C denotes the point of steady-state stress in the ideal DRV-type stress–strain curves. The dashed line represents the linear decline of the work hardening rate *θ* to zero as the flow stress increases from *σ*_c_ to *σ*_p_, as described by Equation (16).

(16) θ=dσ(ε)/dε=kσ(ε)+b

**Figure 16:**
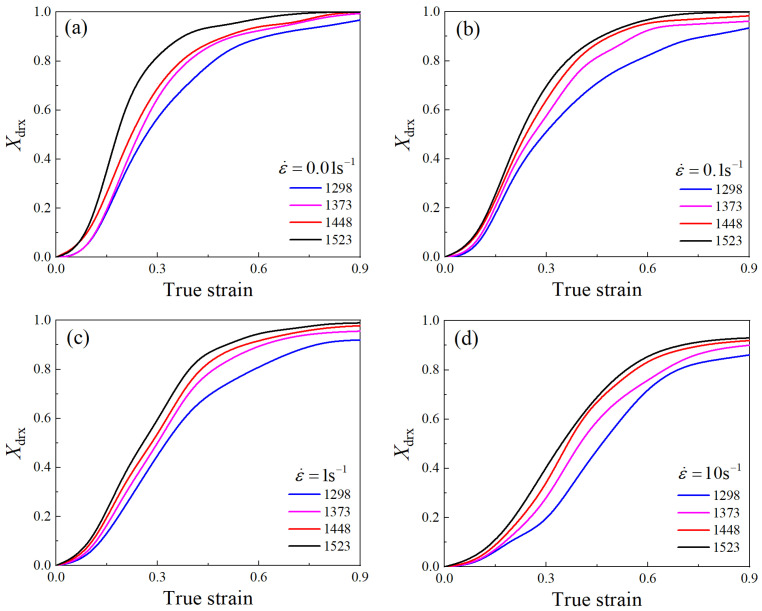
DRX volume fraction under temperatures of 1298–1523 K and strain rates of 0.01–10 s^−1^: (**a**) 0.01 s^−1^, (**b**) 0.1 s^−1^, (**c**) 1 s^−1^, (**d**) 10 s^−1^.

The values *σ*_drvss_ and *σ*_drvx_ can be calculated by substituting *σ*_c_ and its corresponding *θ*_c_ into Equation (16). Therefore, *X*_drx_ values were determined for different deformation conditions, as shown in Figure 16. 

Taking the logarithm on both ends of Equation (13) gives Equation (17).

(17) $$\ln\left[-\ln(1-X_{\mathrm{drx}})\right]=\ln\beta_{d}+k_{d}\ln\left[\left(\varepsilon -\varepsilon_{c}\right)/\varepsilon_{0.5}\right]$$

The values of *ε*_0.5_ for different deformation conditions were listed in Table 6. Furthermore, the *X*_drx_ values corresponding to the true strains of 0.3, 0.6, and 0.9 for different deformation conditions were obtained from Figure 16. Subsequently, the *k*_d_ value was obtained from the mean slope of the linear fitting line of $\ln[-\ln(1-X_{\mathrm{drx}})]$ and $\ln[(\varepsilon -\varepsilon_{c})/\varepsilon_{0.5}]$, which was calculated as 1.4723. By substituting *k*_d_ into Equation (17), the average value of *β*_d_ was calculated to be 0.7068. 

Calculating the logarithms on both ends of Equation (14), Equation (18) is obtained.

(18) $$\ln\varepsilon_{0.5}=\ln a_{2}+n_{2}\ln d_{0}+m_{2}\ln\dot{\varepsilon }+\frac{Q_{2}}{RT}$$

By the nonlinear fitting of *ε*_p_, *d*_0_, $\dot{\varepsilon },$ and *T* in Equation (18), *a*_2_ = 0.0522, *n*_2_ = −0.0018, *m*_2_ = 0.0876, and *Q*_2_ = 20,776.7190 J/mol were calculated.

Consequently, the DRX volume fraction model of the Ni-38Cr-3.8Al alloy can be expressed as Equations (19) and (20).

(19) $$X_{\mathrm{drx}}=1-\exp\left[-0.7068{\left(\frac{\varepsilon -\varepsilon_{c}}{\varepsilon_{0.5}}\right)}^{1.4723}\right]$$

(20) $$\varepsilon_{0.5}=0.0522{d_{0}}^{-0.018}{\dot{\varepsilon }}^{0.0876}\exp(20,776.7190/RT)$$

3. The grain size model of DRX

The relationship between average grain size *d*_A_ and DRX average grain size *d*_drx_ can be expressed by the following Equation (21) [38].

(21) $$d_{A}=X_{\mathrm{drx}}d_{\mathrm{drx}}+(1-X_{\mathrm{drx}})d_{0}$$

where *d*_A_ is the average grain size under various deformation temperatures and strain rates (counted in Table 3), μm, and *d*_drx_ is the DRX average grain size, μm. 

By using Equation (21) in combination with the DRX volume fraction depicted in Figure 16, the DRX grain sizes for various deformation conditions were obtained, as shown in Table 7.

In order to express the connection between the DRX average grain size and the strain rate, the Sellers model is generally utilized, as displayed in Equation (22).

(22) $$d_{\mathrm{drx}}=a_{3}{d_{0}}^{n_{3}}{\dot{\varepsilon }}^{m_{3}}\exp(-Q_{3}/\mathrm{RT})$$

By taking the logarithms of both sides of Equation (22), Equation (23) can be derived.

(23) $$\ln d_{\mathrm{drx}}=\ln a_{3}+n_{3}\ln d_{0}+m_{3}\ln\dot{\varepsilon }-\frac{Q_{3}}{\mathrm{RT}}$$

A nonlinear fit to *d*_drx_, *d*_0_, $\dot{\varepsilon },$ and *T* in Equation (23) yields *a*_3_ = 144.5336, *n*_3_ = 0.7071, *m*_3_ = −0.1159, and *Q*_3_ = 52,478.8461 J/mol. 

By bringing the calculated parameters into Equation (22), the DRX grain size model for the Ni-38Cr-3.8Al alloy can be obtained, as shown in Equation (24).

(24) $$d_{\mathrm{drx}}=144.5336{d_{0}}^{0.7071}{\dot{\varepsilon }}^{-0.1159}\exp(-52,478.8461/\mathrm{RT})$$

### 3.3. Comprehensive Effect of Grain Coarsening and Grain Refinement

**Figure 17:**
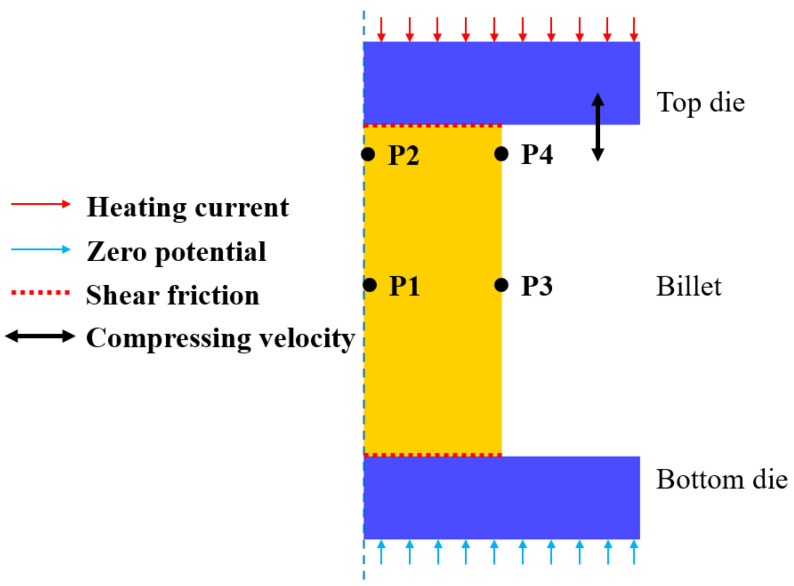
The FE model of isothermal compression.

Based on the results above, an FE model was established in DEFORM V10.2 software to illustrate the comprehensive effect of grain coarsening and grain refinement during thermal compression. The solved kinetics model for grain growth and DRX were integrated into the FE model to simulate microstructure evolution. Figure 17 illustrates the established isothermal compression FE model. In order to improve simulation efficiency, the FE models for thermal compression were simplified into two-dimensional axisymmetric models. The blue dashed line represents the symmetry axis. The dimensions of the workpiece in the FE model matched those in the experiments. Considering the negligible elastic deformation of the workpiece at high temperatures and significant plastic deformations, the workpiece was considered a plastic body, while the top and bottom dies were modeled as rigid bodies. In order to stabilize the deformation temperature of the workpiece, a real-time adjustable heating current was applied to the upper surface of the top die, while a zero potential was applied to the lower surface of the bottom die. The friction between the workpiece and the die was considered as shear friction with a coefficient of 0.3, and the heat transfer coefficient between the workpiece and the die was taken as 0.033. The compressing velocity was imposed on the top die and could be calculated using Equation (25).

(25) $$v=l_{0}\dot{\varepsilon }\exp(-\dot{\varepsilon }t)$$

where *v* is the compressing velocity, *l*_0_ is the initial height of the specimen, and *t* is the deformation time. Points P1–P4 in the billet (seen in Figure 17) were selected to observe the grain size evolution during thermal compression.

#### 3.3.1. Grain Coarsening Effect during Heating and Holding Stages

**Figure 18:**
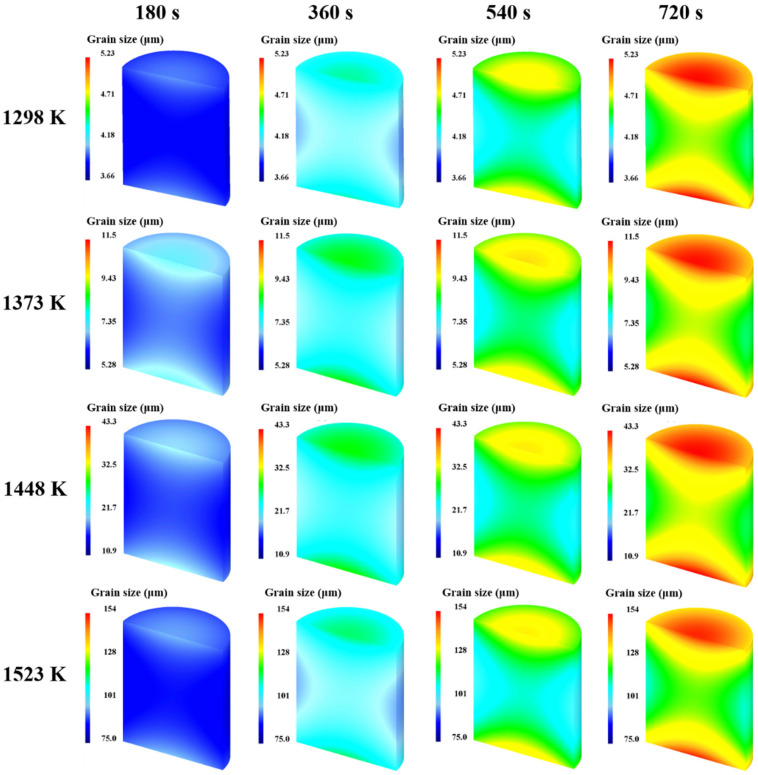
Grain size distributions of the specimens under different holding temperatures and holding times.

**Figure 19:**
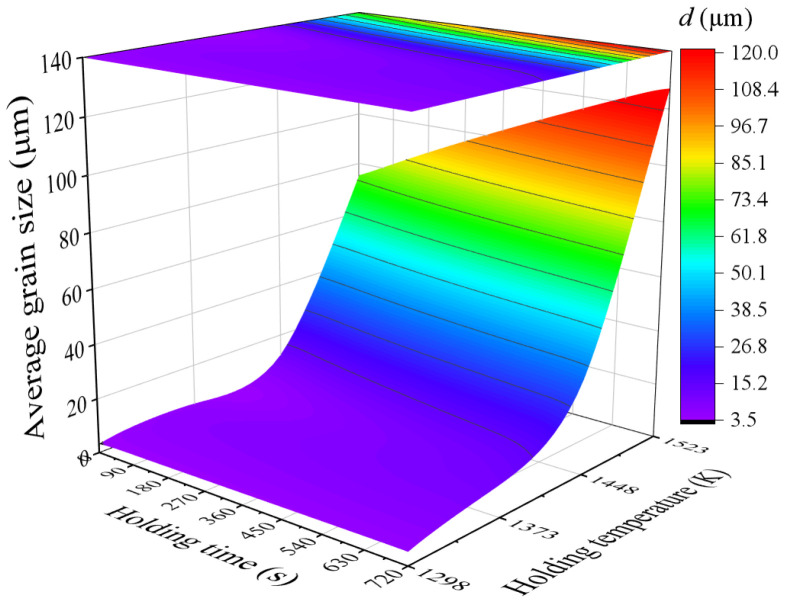
Response surface of average grain size on holding temperature and holding time at point P1.

**Figure 20:**
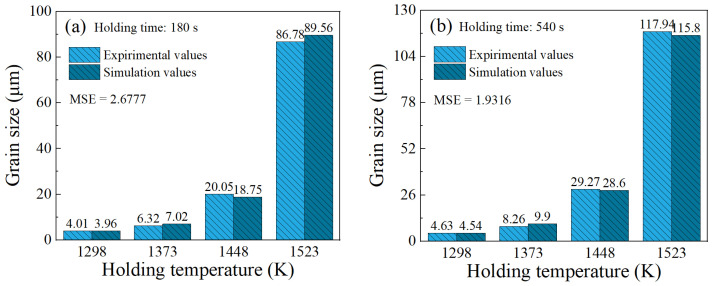
Comparisons of experimental and simulated grain sizes for point P1 under different holding temperatures and holding time: (**a**) holding time: 180 s, (**b**) holding time: 540 s.

The established FE model was employed to simulate the grain size evolution during a holding time of 0–720 s at temperatures between 1298 and 1523 K. The specimen was heated to the specified temperature at a heating rate of 5 K/s. Then, the average temperature of the specimen was stabilized at the specified holding temperature by adjusting the current in real time. Figure 18 illustrates the grain size distributions of the specimens under different holding temperatures and holding times. It can be seen that the grain size distributions in the specimen are uneven. This is due to the heating method used, where the current is applied to the end face of the specimen, causing an uneven temperature distribution inside the specimen. Based on the simulated data, the response surface of the average grain size at point P1 on the holding temperature and holding time was constructed, as depicted in Figure 19. The variation trend of the average grain size depicted by the response surface is consistent with the experimental results. Figure 20 compares the experimental and simulated grain sizes at point P1 under different holding temperatures and holding times, and the results show good agreement. 

#### 3.3.2. Comprehensive Effect during Compression Stage

**Figure 21:**
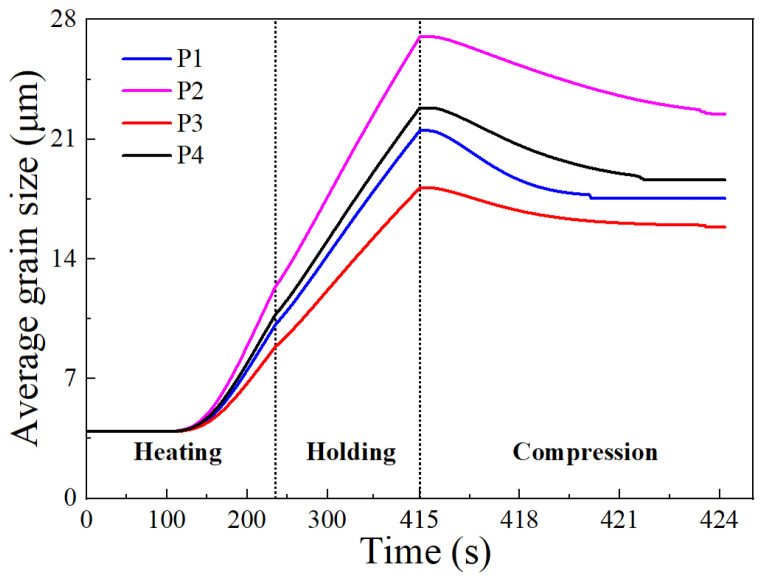
Evolutions of the average grain size at target points during thermal compression process.

A complete thermal compression process was simulated using the developed FE model. In this process, the specimen was heated to 1448 K at 5 K/s, held for 180 s, and then compressed at a strain rate of 0.1 s^−1^. Figure 21 illustrates the evolutions of the average grain size at points P1, P2, P3, and P4 during this process. The trend shows an initial increase to a peak value, followed by a brief plateau, and then a decrease to a minimum value to remain constant. This trend reflects the competition between grain coarsening and grain refinement. During the heating and holding stages, grain growth dominates, leading to a rapid rise in grain size. At the start of the compression, the grain size briefly stabilizes as DRX begins to counteract grain growth. As the compression continues, DRX intensifies, surpassing grain growth, causing a rapid decrease in grain size. Finally, a balance between grain refinement and coarsening is reached, stabilizing the grain size. Notably, the final grain size varies between the four points. This is because the DRX degree is strongly correlated with the amount of deformation, which can differ at various points within the specimen. 

**Figure 22:**
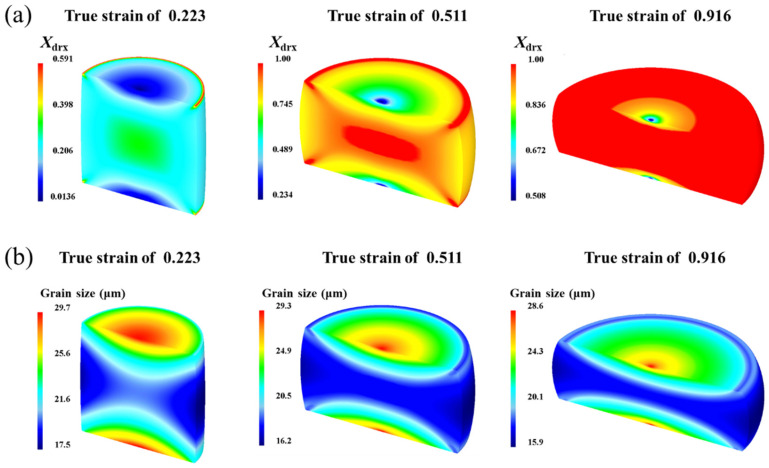
Distributions of the grain size and DRX volume fraction during thermal compression: (**a**) DRX volume fraction distributions, (**b**) grain size distributions.

Figure 22a demonstrates the DRX volume fraction evolution and distribution during the compression stage. With increasing strain, the DRX volume fraction rises, proving that the refinement effect of DRX increases with the strain. The DRX volume fraction distribution varies across the specimen, with the central region exhibiting a significantly higher DRX volume fraction, while the top and bottom faces display lower fractions. The corresponding simulation results of the grain size are shown in Figure 22b. The grain size distribution is uneven throughout the specimen, with the central region having the smallest grain size due to the highest degree of DRX. As the strain increases, grain refinement becomes more pronounced.

**Figure 23:**
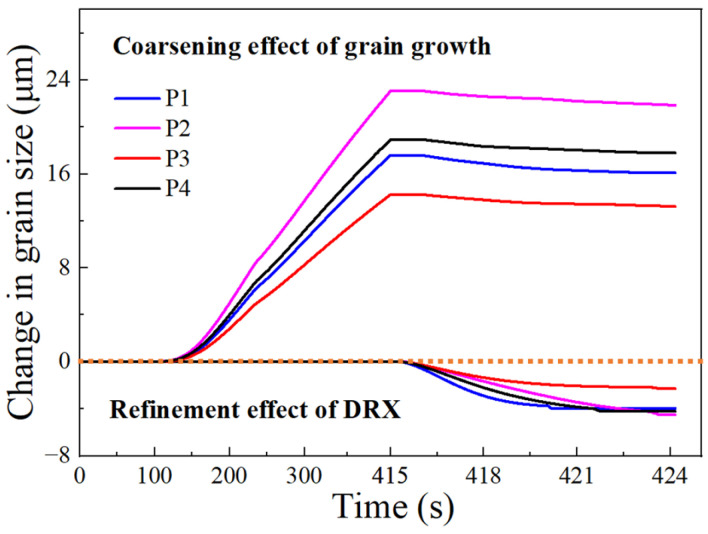
Separative effects of grain growth and DRX at target points during thermal compression process.

Grain growth and DRX behaviors jointly govern the evolution of grain size during the thermal compression process. In order to better understand the competition between grain coarsening and grain refinement in this process, it is crucial to separate the two coexisting mechanisms and quantify their respective impact on grain size. In the heating and holding stages, the grain refinement effect of DRX does not exist, and the grain coarsening effect in these two stages is the difference between the simulated grain size and the initial grain size. In the compression stage, grain refinement and grain coarsening coexist. To quantify the effects of grain refinement and coarsening in this stage separately, two kinds of compression simulation after the holding stage need to be carried out. The first simulation will couple only with the DRX model, while the second one will couple with both the DRX and grain growth models. Then, the grain refinement effect during compression can be quantified by subtracting the grain size at the end of the holding stage from the grain size of the first simulation. And, the grain coarsening effect is the grain size of the second simulation minus the quantized grain refinement effect as well as the initial grain size. By the above method, the grain coarsening and refinement were separated and quantified at points P1–P4 during compression, as shown in Figure 23. It can be seen that grain growth and DRX behaviors alternately dominate during the thermal compression process. In the heating and holding stages, the grain growth behavior dominates. As the compression begins, the coarsening effect starts to weaken, while the refinement effect of DRX gradually increases.

During the compression stage, the deformation parameters significantly affected the comprehensive effect of grain coarsening and grain refinement. Simulations of the thermal compression process with deformation temperatures of 1298–1523 K and strain rates of 0.01–10 s^−1^ were carried out, and the average deformed grain sizes *d*_A_ under different deformation conditions were obtained. In order to quantitatively describe the comprehensive effect during the compression stage under different deformation conditions, the grain refinement ratio (GRR) was introduced, as defined in Equation (26) [23].

(26) $$\mathrm{GRR}=\frac{d_{0}-d_{A}}{d_{0}}$$

where *d*_A_ is the average deformed grain size at point P1 obtained through the simulation, and *d*_0_ is the average grain size at point P1 for the end of the holding stage at different deformation temperatures.

**Figure 24:**
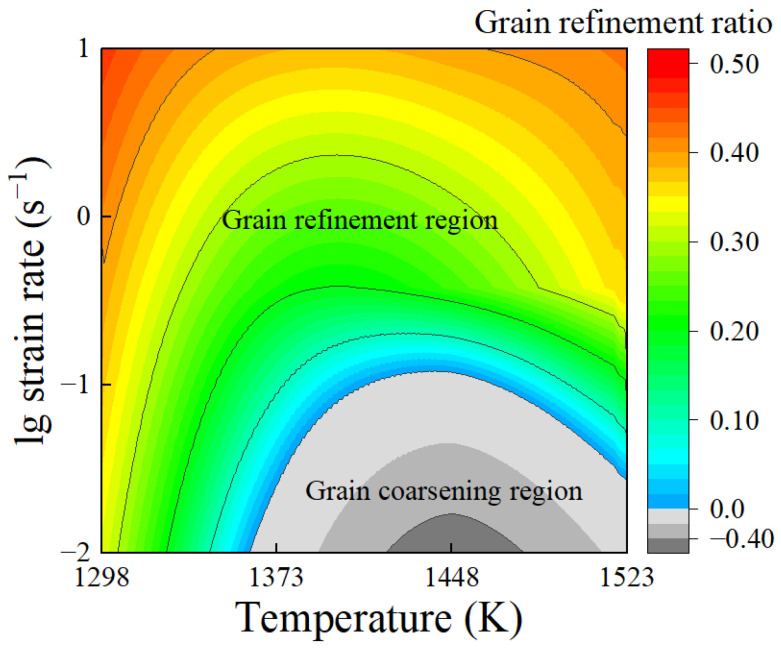
The contour map of grain size refinement ratio.

**Figure 25:**
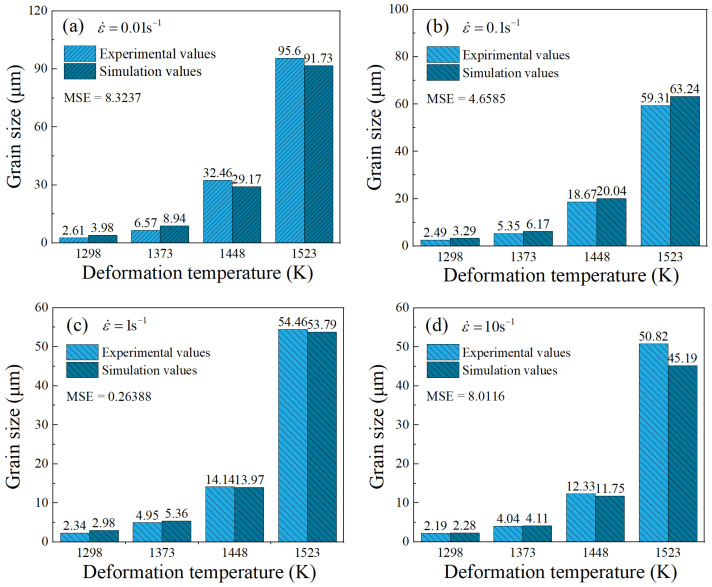
Comparisons of experimental and simulated grain sizes for point P1 under different deformation conditions: (**a**) 0.01 s^−1^, (**b**) 0.1 s^−1^, (**c**) 1 s^−1^, (**d**) 10 s^−1^.

Based on the simulation results, a contour map illustrating the grain refinement ratio was constructed. Figure 24 intuitively exhibits the trends in the grain refinement ratio with variations in the deformation temperature and strain rate. The GRR values greater than 0 are represented by the colored region, which corresponds to the grain refinement region, whereas the gray region represents GRR values less than 0, indicating the grain coarsening region. It has been found that grains generally undergo refinement in the high-strain-rate region (strain rates exceeding 0.1 s^−1^), and the GRR value increases with the rise in strain rates. When the strain rate is below 0.1 s^−1^, grain refinement is observed at deformation temperatures below 1373 K. However, in the deformation temperature range of 1373 K to 1523 K, the grains are coarsened compared to that before deformation. In summary, grain refinement dominates the deformed grain size at higher strain rates (>0.1 s^−1^), while grain coarsening becomes predominant at lower strain rates (<0.1 s^−1^) and higher deformation temperatures (*T* > 1373 K). The comparisons of deformed grain sizes between the experimental results and the simulated ones are presented in Figure 25. The calculated MSE for all data is 5.3144, indicating that the simulation results match the experimental results well. This approves that the developed FE model can accurately characterize the comprehensive effect of grain coarsening and grain refinement throughout the thermal deformation process, enabling the precise prediction of the grain size evolution.

## 4. Conclusions

This paper investigated the separative and comprehensive effects of grain coarsening and grain refinement in the Ni-38Cr-3.8Al alloy through grain growth experiments and isothermal compression tests. An FE model incorporating grain growth and DRX kinetics models was developed to predict the grain size evolution during a thermal compression process. The respective effects of grain growth and DRX on grain size were separated and quantified based on the simulation data. The following conclusions were drawn:

(1) As the holding temperature and time increase, the grain coarsening effect becomes more significant. The grain growth rate increases at higher temperatures but decreases with prolonged holding times. When the temperature exceeds 1400 K, there is a notable acceleration in the grain growth rate.

(2) Under deformation temperatures spanning from 1298 K to 1523 K and strain rates ranging from 0.01 s^−1^ to 10 s^−1^, the compressed specimens exhibit a high degree of DRX. The grain refinement effect intensifies as the deformation temperature decreases and the strain rate increases.

(3) The simulation results closely matched the experimental ones, which showed that grain coarsening predominated during the heating and holding stages. However, during the compression stage, grain refinement dominated at strain rates exceeding 0.1 s^−1^, while grain coarsening dominated at lower strain rates (<0.1 s^−1^) and higher deformation temperatures (>1373 K).

## Figures and Tables

**Table 1:** The average grain sizes under different holding temperatures and holding times.

Temperature (K)	Holding Time (s)			
	0	180	360	540
1298	3.66 ± 0.22 μm	4.01 ± 0.18 μm	4.46 ± 0.19 μm	4.63 ± 0.29 μm
1373	5.28 ± 0.41 μm	6.32 ± 0.58 μm	7.35 ± 0.45 μm	8.26 ± 0.32 μm
1448	10.92 ± 0.77 μm	20.05 ± 1.02 μm	24.26 ± 1.39 μm	29.27 ± 2.83 μm
1523	74.98 ± 2.32 μm	86.78 ± 4.87 μm	107.56 ± 3.57 μm	117.94 ± 5.76 μm

**Table 2:** The *Q*_gg_ values, *A* values, and mean squared errors under different *m* values.

*m*	*Q*_gg_ (J/mol)	*A*	MSE
1	284,400.37	5.28 × 10^8^	6.18
2	507,848.81	3.13 × 10^18^	5.25
3	732,674.24	1.78 × 10^28^	13.46
4	958,696.46	9.12 × 10^37^	21.20
5	1,185,725.65	7.31 × 10^47^	30.90
6	1,413,586.22	5.22 × 10^57^	37.63
7	1,642,126.99	3.94 × 10^67^	43.17
8	1,871,222.67	3.11 × 10^77^	47.80

**Table 3:** The average deformed grain sizes under various temperatures and strain rates.

Temperature (K)	Strain Rate (s^−1^)			
	0.01	0.1	1	10
1298	2.61 ± 0.23 μm	2.49 ± 0.19 μm	2.34 ± 0.10 μm	2.19 ± 0.13 μm
1373	6.57 ± 0.69 μm	5.35 ± 0.34 μm	4.95 ± 0.59 μm	4.04 ± 0.54 μm
1448	32.46 ± 2.12 μm	18.67 ± 2.55 μm	14.14 ± 1.04 μm	12.33 ± 1.21 μm
1523	95.60 ± 5.57 μm	59.31 ± 3.83 μm	54.46 ± 2.83 μm	50.82 ± 3.83 μm

**Table 4:** Values of *ε*_c_ and *ε*_p_ under temperatures of 1298–1523 K and strain rates of 0.01–10 s^−1^.

Strain Rate (s^−1^)	Temperature (K)							
	1298		1373		1448		1523	
	*ε*_c_	*ε*_p_	*ε*_c_	*ε*_p_	*ε*_c_	*ε*_p_	*ε*_c_	*ε*_p_
0.01	0.0153	0.0992	0.0122	0.0938	0.0119	0.0872	0.0105	0.0831
0.1	0.0187	0.1147	0.0169	0.1057	0.0136	0.1024	0.0118	0.0953
1	0.0226	0.1221	0.0181	0.1195	0.0166	0.1117	0.0132	0.0959
10	0.0240	0.1263	0.0199	0.1227	0.0174	0.1134	0.0142	0.1055

**Table 5:** The pre-deformation grain sizes *d*_0_ at different deformation temperatures.

**Temperature (K)**	1298	1373	1448	1523
**Grain size (μm)**	4.01 ± 0.18 μm	6.32 ± 0.58 μm	20.05 ± 1.02 μm	86.78 ± 4.87 μm

**Table 6:** Values of ε_0.5_ under different deformation conditions.

Temperature (K)	Strain Rate (s^−1^)			
	0.01	0.1	1	10
1298	0.25946	0.28919	0.32072	0.45856
1373	0.23784	0.25225	0.2991	0.3964
1448	0.21532	0.23243	0.28378	0.36486
1523	0.16667	0.21532	0.25405	0.34144

**Table 7:** The DRX grain sizes under different temperatures and strain rates.

Temperature (K)	Strain Rate (s^−1^)			
	0.01	0.1	1	10
1298	2.56	2.38	2.19	1.90
1373	6.57	5.31	4.89	3.79
1448	32.46	18.64	14.01	11.67
1523	95.6	59.31	54.12	48.13

## Data Availability

Data are contained within the article.
